# Vitamin D Deficiency among Patients Presenting to Outpatient Department of Medicine of a Tertiary Care Centre: A Descriptive Cross-sectional Study

**DOI:** 10.31729/jnma.7452

**Published:** 2022-05-31

**Authors:** Adhyashree Karki, Shreeju Vaidhya, Dipak Kunwar, Rajyashree Kunwar

**Affiliations:** 1 Department of Gastroenterology and Internal Medicine, KIST Medical College, Gwarko, Kathmandu, Nepal; 2 Department of Nephrology, College of Medical Sciences, Bharatpur, Chitwan, Nepal; 3 Department of Psychiatry, Kathmandu University School of Medical Sciences, Dhulikhel, Kavre, Nepal; 4 Department of HIV and STD control, Save the Children, Airport Gate Area, Kathmandu, Nepal

**Keywords:** *avitaminosis*, *prevalence*, *vitamin deficiency*

## Abstract

**Introduction:**

Vitamin D deficiency is a global health concern with over billions of people worldwide being vitamin D deficient or insufficient. Many epidemiological studies have reported cardiovascular diseases, autoimmune diseases and neoplastic diseases to be associated with vitamin D levels. This study aims to find out the prevalence of vitamin D deficiency in patients presenting to the outpatient Department of Medicine of a tertiary care centre.

**Methods:**

This was a descriptive cross-sectional study done among 362 patients in the outpatient Department of Medicine of a tertiary care centre between May, 2016 and August, 2016. Ethical Approval was taken from Institutional Review Committee (Reference number: 21082015). Convenience sampling was done. Informed consent was obtained and data were collected. Data were analysed using the Statistical Package for the Social Science version 25.0. Point estimate at a 95% Confidence Interval was calculated along with frequency and percentages for binary data.

**Results:**

Out of 362 patients, vitamin D deficiency was found in 215 (59.39%) (54.33-64.45 at 95% Confidence Interval) patients.

**Conclusions:**

The prevalence of vitamin D deficiency was found to be lower to the other studies done in in similar settings. Physicians should be aware of the growing prevalence of vitamin D deficiency.

## INTRODUCTION

Vitamin D is a steroid hormone that helps in the metabolism of minerals, especially calcium, and is essential for healthy bones.^1^ Vitamin D is also important for growing children and adolescents, especially for extraskeletal functions such as improvement of glycaemic control through augmentation of insulin production,^2^ reductions of fasting plasma glucose and insulin resistance,^3^ improvements in cardiovascular function^4^ and both innate and adaptive immune system.^5^

Over billions of people worldwide are vitamin D deficient or insufficient.^6^ Vitamin D levels have been associated with various diseases.^7-9^

The objective of our study is to find out the prevalence of vitamin D deficiency in patients presenting to the outpatient Department of Medicine of a tertiary care centre.

## METHODS

This was a descriptive cross-sectional study done in the Outpatient Department of Medicine of Kathmandu Medical College and Teaching Hospital between May, 2016 to August, 2016. Ethical Approval was taken from the Institutional Review Committee (IRC) (Reference number: 21082015). Convenience sampling was done. Informed consent was obtained once participants agreed to the study. The sample size was calculated using the following formula:

n = (Z^2^ × p × q) / e^2^

  = (1.96^2^ × 0.74 × 0.26) / 0.05^2^

  = 296

Where,

n = minimum required sample size
Z = 1.96 at 95% Confidence Interval (CI)
p = prevalence of vitamin D deficiency, 73.6%^10^
q = 1-p
e = margin of error, 5%

The total sample size is 296. Adding a 10% nonresponse rate, the sample size was found to be 326. However, 362 sample size was taken. Data was collected using a proforma focusing on the sociodemographic profile of patients such as age, marital status, occupation, education, religion, caste, socioeconomic status, personal history of medical illness, alcohol use, and tobacco use.

Vitamin D deficiency was defined as 25(OH)D of <20 ng/ml; vitamin D insufficiency as 25(OH)D of 20 to 29.9 ng/ml and vitamin D sufficiency as 25(OH)D of >30 ng/ ml.^11^ Data were analysed using the Statistical Package for the Social Science version 25.0. Point estimate at a 95% Confidence Interval was calculated along with frequency and percentages for binary data.

## RESULTS

Out of 362 patients, vitamin D deficiency was found in 215 (59.39%) (54.33-64.45 at 95% Confidence Interval) patients. Among them, 117 (54.41%) were below 53 years of age and 98 (48.58%) were below 53 years of age. Vitamin D deficiency was more common among females 112 (52.09%). The sociodemographic details of the patients with vitamin D deficiency are tabulated below (Table 1).

**Table 1:** Sociodemographic profile (n = 215).

Variables		n (%)
Age	<53 years	117 (54.41)
	53 years and above	98 (45.58)
Sex	Males	103 (47.90)
	Females	112 (52.09)
Education	Non-formal	91 (42.32)
	Formal	124 (57.67)
Working status	Unemployed	101 (46.97)
	Employed	114 (53.02)
Alcohol use	Yes	51 (23.72)
	No	164 (76.27)
Tobacco use	Yes	23 (10.69)
	No	191 (88.83)
	Ex-user	1 (0.46)
BMI	<25	88 (40.93)
	25 and above	127 (59.06)

One hundred fifteen (53.48%) patients had hypertension and 55 (25.58%) had diabetes mellitus (Table 2).

**Table 2:** Vitamin D deficiency in patient with comorbidities (n = 215).

Variables		n (%)
Duration of hypertension	Up to 8 years	75 (34.88)
	8 years and above	140 (65.11)
Duration of diabetes mellitus	Up to 9 years	37 (17.20)
	9 years and above	178 (82.79)
Thyroid function test	Normal	147 (68.37)
	Hyperthyroidism	14 (6.51)
	Hypothyroidism	54 (25.11)

## DISCUSSION

There is ample of evidence to prove that vitamin D deficiency is responsible for multiple chronic conditions like diabetes mellitus, hypertension, cardiovascular disease, and several other autoimmune disorders.^9^ With the growing prevalence of vitamin D deficiency across the world, it has become more important than ever to study the prevalence of vitamin D and to pinpoint the risk factors for vitamin D deficiency.

The main finding from our study was that 59.39% had vitamin D levels less than 20 ng/ml. The study from Nepal reported the prevalence of vitamin D deficiency was 73.6% among patients presenting to tertiary care centre which is found to be slighty higher than our study.^10^ A study reported that vitamin D levels lower than 20 ng/ml were seen in about 30% to 50% of children and adults, in the United Arab Emirates, Australia, Turkey, India and Lebanon.^12^

Various studies from Nepal have shown similar findings. A retrospective study was done in Star Hospital, Sanepa with a total of 786 patients whose vitamin D levels were tested showed 717 (91.2%) had deficiency which is higher than the finding of our study. Adult females were found to be vitamin D deficient than the adult male population whose finding is similar to that of this study.^13^

In a study conducted at Manipal College of Medical Sciences, the mean vitamin D level was low in the age group above 60 years as compared to other age group patients. Prevalence of low levels of vitamin D was seen more in females (90.3%) as compared to males (68.0%).^14^

A study done by showed diabetes mellitus was seen present as a comorbodity with vitamin deficiency which is in agreement with this study.^15^ One-fourth of the patient with Vitamin D deficiency in our study had hypothyroidism. Vitamin D deficiency is usually associated with hypothyroidism. Many studies supported this hypothesis.^16^

A cross-sectional study conducted in Kathmandu Medical College reported out of total of 384 patients, vitamin D deficiency was found among 283 (73.6%) patients at 95% of CI (68.6-78.6). Out of total female patients, 202 (52.61%) were deficient and out of total male patients, 81 (21.08%) were deficient which is similar to the findings of this study as 178 (82.8) with diabetes of longer duration than 9 years had vitamin D deficiency.^10^

A cross-sectional study done among 2158 patients in western Nepal showed that 1590 (73.68%) had vitamin D deficiency, whereas only 568 (26.32%) had optimum level of vitamin D. Females were more deficient than male by 5.29% which is similar to this study.^17^ Similarly, a cross-sectional study based on the hospital registry of patients at College of Medical Sciences and Teaching Hospital, Bharatpur showed higher level of vitamin D deficiency in the females than in the males (72.4% vs 64.2%) which is similar to the finding of this study.^18^

There has been several limitations in our study. First, as it was a cross-sectional study, we could not establish a causal relationship based on the results. The participants were regular patients presenting in the general medicine OPD, so the result could not be compared to the general population.

## CONCLUSIONS

The prevalence of vitamin D deficiency was found to be lower to the other studies done in in similar settings. Physicians should be aware of the growing prevalence of vitamin D deficiency not only among patients and more research needs to be conducted to pinpoint the effects of socio-demographic factors on vitamin D deficiency. More research needs to be conducted to pinpoint the effects of socio-demographic factors on Vitamin D deficiency.
